# TIMP-3和mtp53在非小细胞肺癌中的表达及意义

**DOI:** 10.3779/j.issn.1009-3419.2012.04.02

**Published:** 2012-04-20

**Authors:** 红 杨, 婕 李, 嘉 郭, 尚福 张

**Affiliations:** 1 610041 成都，四川大学华西医院病理科 Department of Pathology, West China Hospital, Sichuan University, Chengdu 610041, China; 2 610041 成都，四川省肿瘤医院病理科 Department of Pathology, Sichuan Cancer Hospital, Chengdu 610041, China; 3 611930 彭州，彭州市人民医院病理科 Department of Pathology, People's Hospital of Pengzhou City, Pengzhou 611930, China

**Keywords:** 肺肿瘤, TIMP-3, mtp53, 组织芯片, 预后, Lung neoplasms, TIMP-3, mtp53, Tissue microarray, Prognosis

## Abstract

**背景与目的:**

基质金属蛋白酶组织抑制剂-3（tissue inhibitor of metalloproteinases-3, TIMP-3）可通过多种途径调节肿瘤的浸润和转移，且可能与突变型*p53*（mutant-type *p53*, mtp53）存在一定的相关性。本研究旨在利用组织芯片技术检测TIMP-3和mtp53在非小细胞肺癌（non-small cell lung cancer, NSCLC）组织及其淋巴结转移癌中的表达并探讨其意义。

**方法:**

应用免疫组织化学LSAB法和Elivision法检测24例良性病变肺支气管黏膜上皮组织（对照组）、288例NSCLC组织（原发灶）及106例淋巴结转移灶癌组织（转移灶）中TIMP-3和mtp53的表达。

**结果:**

原发灶与转移灶中TIMP-3的表达明显低于对照组（*P* < 0.001），而mtp53的表达明显高于对照组（*P* < 0.001）；TIMP-3和mtp53在伴有/不伴有淋巴结转移的原发灶中的表达差异有统计学意义（*P*=0.015, *P*=0.030）。TIMP-3的表达与NSCLC的病理学分级有关（*P*=0.030），mtp53的表达与NSCLC的TNM分期和组织学类型有关（*P*=0.016, *P*=0.004）。TIMP-3与mtp53在原发灶中的表达呈负相关（*P*=0.008），其表达均与NSCLC患者的术后生存率有关（*P*=0.011, *P*=0.003）。

**结论:**

TIMP-3的低表达和mtp53的高表达都可促进肺癌的转移，且二者在肺癌转移中相互抑制，可能成为研究NSCLC转移机制的新靶点。

## Abstract (en)

**Background and objective:**

Tissue inhibitor of metalloproteinase-3 (TIMP-3) can regulate tumor infiltration and metastasis through multiple channels and is likely associated with mutant-type *p53* (mtp53). This study detected the expressions of TIMP-3 and mtp53 in non-small cell lung cancer (NSCLC) and lymph node metastasis using tissue microarray and evaluated their significance.

**Methods:**

TIMP-3 and mtp53 expressions were detected in 288 cases of NSCLC (NSCLC group), 106 cases of metastatic carcinoma in lymph nodes (metastasis group), and 24 cases of benign lesions in the bronchial mucosa epithelium (control group) by immunohistochemical staining (LSAB and Elivision).

**Results:**

The expression of TIMP-3 in the NSCLC and metastasis groups was lower than that in the control group (*P* < 0.001), but the reverse was true for the expression of mtp53 (*P* < 0.001). TIMP-3 and mtp53 expressions differed between NSCLC with (*P*=0.015) and without (*P*=0.030) lymph node metastasis. TIMP-3 expression correlated with NSCLC grade (*P*=0.030), whereas mtp53 expression correlated with TNM stage (*P*=0.016) and NSCLC histological type (*P*=0.004). Moreover, the expressions of TIMP-3 and mtp53 were negative in NSCLC cases (*P*=0.008) and correlated with patient survival (*P*=0.011 and *P*=0.003, respectively).

**Conclusion:**

Low expression of TIMP-3 and high expression of mtp53 in NSCLC can promote tumor metastasis and inhibit each other. TIMP-3 and mtp53 are promising targets for studying the metastatic mechanism of NSCLC.

肺癌是人类最常见的恶性肿瘤之一，患者的5年和10年生存率分别为14%和8%^[1]^。近年来肺癌的诊断和治疗技术取得了一定进步，但肺癌患者的生存率并未明显提高。肺癌常在早中期发生邻近组织、器官的浸润及淋巴结转移，是其治疗失败的主要原因。基质金属蛋白酶组织抑制剂-3(tissue inhibitor of metalloproteinases-3, TIMP-3)在抑制肿瘤的浸润和转移方面发挥作用，且与突变型*p53*(mutant-type *p53*, mtp53)存在一定的相关性。本研究利用组织芯片技术和免疫组织化学染色方法检测TIMP-3和mtp53在非小细胞肺癌(non-small cell lung cancer, NSCLC)组织及其淋巴结转移灶中的表达情况，探讨它们与NSCLC的转移、组织学类型、病理分级、TNM分期和预后等方面的关系以及二者之间的相关性，为临床预防肿瘤转移的治疗措施提供可能的依据。

## 1 材料与方法

### 1.1 材料

收集四川大学华西医院1998年1月-2001年12月接受根治性手术治疗且保存有完整临床资料和石蜡组织块的NSCLC病例，按照2003年WHO肺肿瘤组织学分类的标准，对其组织学类型、病理分级、有/无转移进行评判。共筛选出原发性NSCLC 288例，男性223例，女性65例，平均年龄59.5岁(29岁-82岁)；鳞状细胞癌(以下简称为鳞癌)125例，腺癌129例，腺鳞癌34例；高分化癌27例，中分化癌112例，低分化癌115例(34例腺鳞癌无法进行分级)；伴有转移的原发灶肺癌组织150例，实际收集其淋巴结转移灶癌组织106例。根据2007年肺癌国际TNM分期修订版标准：Ⅰ期98例，Ⅱ期53例，Ⅲ期102例，Ⅳ期35例。选取同期手术的24例良性病变(包括肺结核、囊肿、炎性假瘤及错构瘤)肺支气管粘膜上皮组织作为对照。患者术前均未行放疗或化疗，并行术后随访，截止日期为2006年8月31日，中位生存时间29个月。生存期的计算从诊断日期起至随访日期或由于复发、转移而死亡的日期为止。

### 1.2 组织芯片的制作

本实验采用手工制作组织芯片。首先选择所需病例的存档蜡块，根据HE染色切片进行形态学观察，确定具有代表性的病变部位(避开出血、坏死、明显炎细胞浸润及纤维化区域)后在HE切片和相应石蜡组织块上标记。根据样本的数量及所要求组织片的大小在空白石蜡块(组织芯片专用)上钻孔，此实验选用钻头的直径为1.5 mm，再用金属空心管(内径仍为1.5 mm)从经定位的目标石蜡块上钻取组织并转移至已钻孔的空白石蜡块的相应位置。每一个选中蜡块(包括原发灶癌组织、转移灶癌组织和对照组织)选3个组织芯放入芯片中(对有可能掉片的病例选4个组织芯放入芯片中)。使用病理切片机制备4 μm组织切片，置于经防脱片处理的载玻片上，放入56 ℃烤箱中烘烤48 h。常温保存备用。

### 1.3 免疫组织化学染色

TIMP-3为兔抗人多克隆抗体(购自博士德生物技术开发有限公司)，工作稀释度为1:100，用微波枸橼酸进行抗原修复，以胎盘绒毛作为阳性对照，采用LSAB法染色(SP法，试剂盒购自北京中山金桥生物技术开发有限公司)。mtp53抗体为鼠抗人单克隆抗体(购自福州迈新生物技术开发有限公司)，工作稀释度为1:50，用水浴EDTA进行抗原修复，以乳腺癌作为阳性对照，采用Elivision法染色(试剂盒购自Dako生物技术开发公司)。

### 1.4 免疫组织化学结果评定

TIMP-3以细胞质内出现棕黄色颗粒为阳性，mtp53以细胞核内出现棕黄色颗粒为阳性。将芯片内所有癌细胞都纳入计数范围内，将着色强度高于背景的细胞定为阳性细胞。细胞质和细胞核内的棕黄色颗粒都分别根据阳性细胞的染色强度(未着色：0分；淡黄色：1分；棕黄色：2分；棕褐色：3分)和阳性细胞所占的构成比评分(全阴：0分；≤10%：1分；11%-50%：2分；51%-75%：3分； > 75%：4分)；将每例获得的两组分数相乘：0-3分为(-)；4分-7分为(+)；8分-12分为(++)^[2]^。

### 1.5 统计学处理

使用SPSS 13.00统计软件进行数据处理，采用卡方检验、*Spearman*等级相关分析、*Log-rank*检验等统计分析方法。*P* < 0.05为差异有统计学意义。

## 2 结果

### 2.1 TIMP-3和mtp53在良性病变、NSCLC原发灶癌和淋巴结转移灶癌中的表达情况

TIMP-3在24例肺良性病变的支气管粘膜上皮细胞中均呈阳性表达(100%)；288例NSCLC原发灶肺癌组织中呈(-)表达42例(14.6%)、(+)表达101例(35.1%)、(++)表达145例(50.3%)，其阳性表达率为85.4%；106例淋巴结转移灶癌组织中呈(-)表达25例(23.6%)、(+)表达38例(35.8%)、(++)表达43例(40.6%)，其阳性表达率为76.4%。原发灶与淋巴结转移灶癌组织TIMP-3的表达明显低于正常支气管黏膜上皮组织(*P* < 0.01)。见图 1。

**1:**
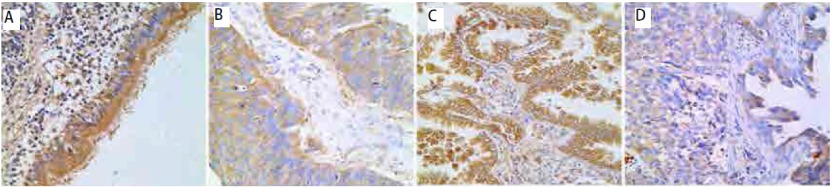
TIMP-3在肺良性病变支气管粘膜上皮组织（A）、鳞状细胞癌（B）、腺癌（C）与腺鳞癌（D）中的表达（LSAB，×400） The expression of TIMP-3 in benign lesion's bronchus mucosa epithelium (A), squamous cell carcinoma (B), adenocarcinoma (C) and adenosquamous carcinoma (D) (LSAB, ×400)

mtp53在24例肺良性病变的支气管粘膜上皮细胞中均呈阴性表达；288例NSCLC原发灶癌组织中呈(-)表达161例(55.9%)、(+)表达54例(18.8%)、(++)表达73例(25.3%)，其阳性表达率为44.1%；在106例淋巴结转移灶癌组织中呈(-)表达53例(50.0%)、(+)表达27例(25.5%)、(++)表达26例(24.5%)，其阳性表达率为50.0%。原发灶与淋巴结转移灶癌组织mtp53的表达明显高于正常支气管粘膜上皮组织(*P* < 0.01)。见图 2。

**2:**
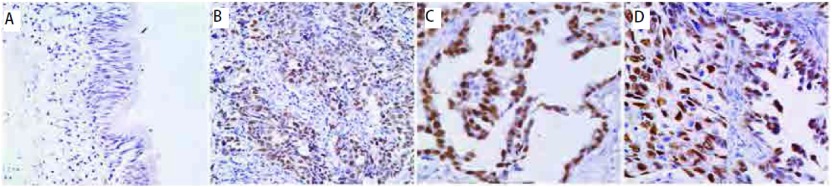
mtp53在肺良性病变支气管黏膜上皮组织（A）、鳞状细胞癌（B）、腺癌（C）与腺鳞癌（D）中的表达（Elivision，×400） The expression of mtp53 in benign lesion's bronchus mucosa epithelium (A), squamous cell carcinoma (B), adenocarcinoma (C) and adenosquamous carcinoma (D) (Elivision, ×400)

### 2.2 TIMP-3和mtp53的表达与转移的关系

不伴有淋巴结转移的138例原发灶癌组织中TIMP-3呈(-)表达14例(10.1%)、(+)表达43例(31.2%)、(++)表达81例(58.7%)，而伴有淋巴结转移的150例原发灶癌组织中TIMP-3呈(-)表达28例(18.6%)、(+)表达58例(38.7%)、(++)表达64例(42.7%)，两组表达差异有统计学意义(*P*=0.015)。不伴有淋巴结转移的138例原发灶癌组织中mtp53呈(-)表达88例(63.8.1%)、(+)表达23例(16.7%)、(++)表达27例(19.5%)，而伴有淋巴结转移的150例原发灶癌组织中mtp53呈(-)表达73例(48.6%)、(+)表达31例(20.7%)、(++)表达46例(30.7%)，两组表达差异有统计学意义(*P*=0.030)。

### 2.3 TIMP-3和mtp53的表达与病理学分级、组织学类型及TNM分期之间的关系

见表 1。TIMP-3的表达与NSCLC的病理学分级有关，在高分化癌细胞中的表达高于中分化和低分化癌细胞，差异有统计学意义(*P*=0.030)；TIMP-3的表达与NSCLC的组织学类型和TNM分期无关(*P* > 0.05)。mtp53的表达与NSCLC的组织学类型和TNM分期有关(*P*=0.004, *P*=0.016)，其在腺癌中的表达最低，在腺鳞癌中的表达最高，随TNM分期的增加而增加，与NSCLC的病理学分级无关(*P*=0.347)。

**1:** TIMP-3和mtp53在不同非小细胞肺癌病变组织中表达差异的分析 Analysis of expression difference of TIMP-3 and mtp53 in distinct non-small cell lung cancer (NSCLC) pathological tissues

Characteristics	*n*	Expression of TIMP-3			*P*	Expression of mtp53			*P*
		(-)	(+)	(++)		(-)	(+)	(++)	
Histological type					0.151				0.004
Squamous cell carcinoma	125	19	48	58		55	33	37	
Adenocarcinoma	129	14	44	71		87	15	27	
Adenosquamous carcinoma	34	9	9	16		19	6	9	
Grade					0.030				0.347
Well	27	1	8	18		20	4	3	
Moderate	112	11	47	54		62	22	28	
Poor	115	21	37	57		60	22	33	
TNM stage					0.148				0.016
Ⅰ	98	11	28	59		66	13	19	
Ⅱ	53	6	19	28		32	10	11	
Ⅲ	102	17	39	46		48	26	28	
Ⅳ	35	8	15	12		15	5	15	
TIMP-3: tissue inhibitor of metalloproteinases-3; mtp53: mutant-type p53.									

### 2.4 TIMP-3和mtp53表达的相关分析

*Spearman*等级相关分析显示TIMP-3与mtp53在NSCLC癌组织中的表达呈负相关(*r*=-0.156, *P*=0.008)。

### 2.5 TIMP-3和mtp53的表达与患者生存的关系

分析TIMP-3和mtp53在NSCLC病灶中的表达与患者的术后生存率有关：TIMP-3在病灶中呈(++)表达的NSCLC患者生存率最高，呈(-)表达者生存率最低，差异有统计学意义(*χ*^2^=9.055, *P*=0.011)；mtp53呈(-)表达的NSCLC患者生存率最高，呈(++)表达者生存率最低，差异有统计学意义(*χ*^2^=11.829, *P*=0.003)(图 3)。

**3:**
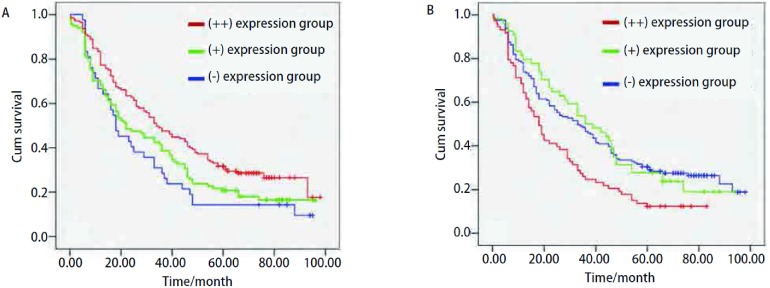
TIMP-3（A）与mtp53（B）在NSCLC患者中不同表达情况的Kaplan-Meier生存曲线 *Kaplan-Meier* survival curves of NSCLC patients with expression of TIMP-3 (A) and mtp53 (B)

## 3 讨论

TIMPs能特异性的抑制基质金属蛋白酶(matrix metalloproteinases, MMPs)的活性，阻止细胞外基质的降解，从而抑制肿瘤的转移。大量研究显示TIMP-3的表达与肿瘤的预后有很大的相关性，Powe等^[3]^研究发现在结肠低分化腺癌的浸润边缘TIMP-3的表达明显降低，提示边缘TIMP-3表达的降低与肿瘤浸润有很大的关系；TIMP-3的表达降低是食管癌的浸润增强、分期增加和预后较差的主要原因^[4, 5]^；Span等^[6]^发现在乳腺癌中，TIMP-3高表达的患者比低表达者预后好，Celebiler等^[7]^的研究结果也显示TIMP-3的低表达促进了乳腺癌转移；Mino等^[8]^研究TIMP-3在肺癌中的表达发现，TIMP-3的弱、中、强表达率差异在鳞癌中表现更为明显，TIMP-3的低表达与淋巴结的转移相关，TIMP-3与患者的生存预后相关。这些研究结果都表明TIMP-3表达的降低会导致肿瘤更具有浸润和转移的能力，而Riddick等^[9]^在前列腺癌中发现TIMP-3的表达与预后没有明显的相关性，而高表达与淋巴结的转移相关，但是在局部还是有抑制肿瘤生长的作用；Kornfeld等^[10]^的研究结果显示在头颈部的鳞状细胞癌中TIMP-3 mRNA高表达者预后较低表达者更差。总之，TIMP-3在不同肿瘤中的作用机制还不是很一致，还需要大量的研究证明。本实验发现TIMP-3在24例肺良性病变的支气管粘膜上皮细胞中均呈100%表达，而在288例NSCLC原发灶癌组织中阳性表达已降为85.4%；且在伴有淋巴结转移的原发灶癌组织中TIMP-3表达明显低于不伴有淋巴结转移的原发灶。因此我们推测TIMP-3的低表达与NSCLC的淋巴结转移有关，这与Mino等^[8]^的研究结果一致。本研究结果中TIMP-3的表达与NSCLC的TNM分期无关，推测主要受肿瘤大小影响。本实验还发现TIMP-3的表达与NSCLC的分化程度有关，NSCLC分化越好，TIMP-3的表达率越高；但TIMP-3的表达与NSCLC的组织学类型无关，这与Mino等^[8]^研究结果不一致，可能由于人种和地域的差异，这方面需要更进一步的研究和分析。

肺癌患者*p53*基因突变率居人类肿瘤的首位，其基因功能的失活在肺癌的发生发展中起着重要作用，60%的NSCLC和80%的小细胞肺癌(small cell lung cancer, SCLC)可检测到*p53*基因的突变^[11]^。张霞等^[12]^研究表明mtp53蛋白在肺癌中表达率为51.8%，其表达率与肿瘤的分化程度有关，分化越差，表达率越高。李爱武等^[13]^研究发现，NSCLC癌组织的mtp53蛋白表达(60%)较正常肺组织(5%)明显增高，提示p53突变可能参与了肺癌的发生发展过程，其中mtp53蛋白在肺腺癌组织中的表达分别高于腺鳞癌及鳞癌，原发肿瘤T3-T4组织中的mtp53蛋白表达较原发肿瘤T1-T2高，mtp53蛋白在低分化癌组织中的表达较中、高分化癌组织高，说明mtp53蛋白的表达与肿瘤的病理学类型、原发肿瘤范围及分化程度有关。本实验结果中mtp53在24例肺良性病变的支气管粘膜上皮细胞中均不表达，而在288例NSCLC原发灶癌组织中阳性表达为44.1%；且在伴有淋巴结转移的原发灶癌组织中mtp53表达明显高于不伴有淋巴结转移的原发灶；同时mtp53的表达随NSCLC的TNM分期增加而升高。本研究中mtp53的表达在腺癌中最低，其次为鳞癌，在腺鳞癌中表达最高，因此mtp53的表达与NSCLC组织学类型的关系还有待于进一步研究。

关于TIMP-3和mtp53的相关性在肺癌的发生、转移和预后中的研究甚少。Thomas^[14]^和Loging等^[15]^发现mtp53具有抑制TIMP-3表达的作用，从而使受TIMP-3抑制的MMPs表达增加，达到抑制肿瘤细胞凋亡和促进肿瘤组织内血管形成的作用，促进肿瘤的生长、浸润与转移。Zalcenstein等^[16]^通过体外实验发现mtp53可以通过抑制Fas启动子的活性，下调Fas mRNA的转录来抑制Fas的表达，从而进一步抑制了TIMP-3通过Fas途径诱导细胞的凋亡。本研究结果也显示，TIMP-3与mtp53在NSCLC组织中的表达存在负相关，与文献报道一致，但其分子机制需要进一步的研究。

综上，TIMP-3的低表达与mtp53的高表达都可以促进NSCLC的转移且二者之间存在抑制作用，可以考虑从增加TIMP-3的表达、降低mtp53的表达方面着手研究更好的肿瘤治疗方法。临床上也可将二者结合起来，有助于提高对NSCLC发生转移可能性和预后等方面预测的准确性，或可能成为NSCLC患者预防肿瘤转移的一种治疗措施。
